# Evaluation of User Satisfaction in a Supportive Text Message Program for Public Safety Personnel

**DOI:** 10.1192/j.eurpsy.2024.1132

**Published:** 2024-08-27

**Authors:** G. Obuobi-Donkor, R. Shalaby, B. Agyapong, R. D. L. Dias, V. I. O. Agyapong

**Affiliations:** ^1^Department of Psychiatry, Dalhousie University, Halifax; ^2^Department of Psychiatry, University of Alberta, Edmonton, Canada

## Abstract

**Introduction:**

Public safety personnel (PSP) encounter traumatic events in their workplace, elevating the likelihood of mental health issues. Delivering efficient, evidence-backed interventions, like supportive SMS text messaging programs, can significantly enhance PSPs’ mental well-being, garnering high user satisfaction rates.

**Objectives:**

This study evaluates users’ satisfaction, receptiveness, and perceptions of the supportive SMS text messaging intervention (Text4PTSI).

**Methods:**

Participants enrolled in the Text4PTSI program and received one-way cognitive behavioural–based supportive text messages for six months. They participated in a web-based survey delivered through SMS text messages at enrollment, six weeks, three months, and six months after enrollment. The participants’ perceptions and receptiveness of the program were evaluated through a 5-point Likert scale. Data were represented as categorical variables, and overall satisfaction with the Text4PTSI program was assessed on a scale ranging from 0 to 100.

**Results:**

Of the 131 Text4PTSI program subscribers, 81 participants responded to the survey, yielding 100 responses across the three follow-up time points. The average satisfaction score was 85.12 (SD 13.35). A significant portion of respondents, constituting 79%, agreed or strongly agreed that Text4PTSI helped them manage anxiety. Additionally, 72% reported relief from depressive symptoms, and 54% (54 out of 100 responses) felt less lonely. Moreover, the majority (84%) of participants expressed that Text4PTSI connected them to a support system, improving their mental well-being, felt more hopeful about managing concerns about their mental health or substance use (82%), and helped enhance their overall quality of life (77%). The data also revealed that most participants consistently read the supportive text messages (84 out of 100 responses, 84%), took time to contemplate each message (75 out of 100 responses, 75%), and revisited the messages more than once (76 out of 100 responses, 76%).

**Conclusions:**

PSP participating in the 6-month Text4PTSI intervention expressed significant satisfaction and gratitude in the follow-up surveys. Their positive feedback indicates a promising path towards increased service utilization, potentially enhancing its effectiveness and impact on end users.

**Disclosure of Interest:**

None Declared

